# Psycholinguistic variables matter in odor naming

**DOI:** 10.3758/s13421-017-0785-1

**Published:** 2018-02-12

**Authors:** John L. A. Huisman, Asifa Majid

**Affiliations:** 1 Centre for Language Studies, Radboud University, Nijmegen, The Netherlands; 2 Centre for Language Studies, and Donders Institute for Brain, Cognition, and Behaviour, Radboud University, P.O. Box 9103, 6500 HB Nijmegen, The Netherlands; 3 Max Planck Institute for Psycholinguistics, Nijmegen, The Netherlands

**Keywords:** Olfaction, Olfactory naming, Word frequency, Semantic interference

## Abstract

People from Western societies generally find it difficult to name odors. In trying to explain this, the olfactory literature has proposed several theories that focus heavily on properties of the odor itself but rarely discuss properties of the label used to describe it. However, recent studies show speakers of languages with dedicated smell lexicons can name odors with relative ease. Has the role of the lexicon been overlooked in the olfactory literature? Word production studies show properties of the label, such as word frequency and semantic context, influence naming; but this field of research focuses heavily on the visual domain. The current study combines methods from both fields to investigate word production for olfaction in two experiments. In the first experiment, participants named odors whose veridical labels were either high-frequency or low-frequency words in Dutch, and we found that odors with high-frequency labels were named correctly more often. In the second experiment, edibility was used for manipulating semantic context in search of a semantic interference effect, presenting the odors in blocks of edible and inedible odor source objects to half of the participants. While no evidence was found for a semantic interference effect, an effect of word frequency was again present. Our results demonstrate psycholinguistic variables—such as word frequency—are relevant for olfactory naming, and may, in part, explain why it is difficult to name odors in certain languages. Olfactory researchers cannot afford to ignore properties of an odor’s label.

We all recognize the smell of freshly baked bread when walking past a bakery, and so you might mistakenly think you can also easily name that odor. However, naming smells in the absence of contextual cues appears to be difficult: on average, people only name 25% to 50% of odors correctly (Cain, 1979, 1982; Desor & Beauchamp, 1974; Distel & Hudson, 2001; Yeshurun & Sobel, 2010), which stands in sharp contrast to the near-ceiling performance reported in visual naming studies (Damian, Vigliocco, & Levelt, 2001; Jescheniak & Levelt, 1994). So, why are people so bad at naming odors?

Various proposals abound (see Jönsson & Stevenson, 2014, for an overview). Perhaps we are not very good smellers because of the loss of functional olfactory receptor genes in modern humans (Gilad, Man, Pääbo, & Lancet, 2003), for example. However, behavioral studies paint a different picture (Majid, Speed, Croijmans, & Arshamian, 2017). Cross-species comparisons show that humans have comparable olfactory sensitivity to nonhuman primates (Laska, Seibt, & Weber, 2000; see also Shepherd, 2004; McGann, 2017). Others argue the problem lies in the link between olfactory and verbal areas of the brain: These links are either inherently weak (e.g., Engen, 1987), interfere with each other (Lorig, 1999), or are too direct (Olofsson & Gottfried, 2015). However, it is unclear whether the nature of this link is the cause of poor odor naming or whether it merely reflects learning history (Majid, 2015). Recently, Majid and Burenhult (2014) showed that Jahai speakers from the Malay Peninsula were able to name odors with more ease than English speakers. Unlike English speakers, Jahai speakers have a dedicated vocabulary to describe different qualities of smell (Burenhult & Majid, 2011). This raises the question of whether poor odor naming could be the result of properties of the lexicon itself; and more broadly, what role psycholinguistic variables play in odor naming.

Prior research examining odor naming has overlooked the role of the lexicon, and focused almost exclusively on properties of the odor instead. It has been shown that an odor’s familiarity is an important factor in free naming (Lawless, 1978; Lawless & Cain, 1975), as well as in recognition (Rabin & Cain, 1984) and discrimination (Rabin, 1988). Odor pleasantness and intensity also influence naming (Distel & Hudson, 2001), with identified odors rated as more intense and pleasant. “Real” odors (i.e., taken from a natural source; e.g., actual chocolate) are named correctly more often than synthetic odors (e.g., microencapsulated odors; cf. Engen, 1987).

In contrast, there is little examination of whether properties of the odor label might impact odor naming, even though some words are simply easier to produce than others. One property shown to influence word production is how often a word is used, that is, its frequency. Pictures with high-frequency labels are named faster (e.g., Oldfield & Wingfield, 1965), and more accurately (e.g., Jescheniak & Levelt, 1994), than pictures with low-frequency labels. Odor-naming studies never control for this factor. It is simply assumed—without ever checking any corpora for the linguistic facts—that because odors used in naming studies are familiar, they must have high-frequency names (cf. Jönsson, 2005). When word frequency has been discussed, it has been used only as a proxy for odor frequency (e.g., Cain et al., 1995; Wijk & Cain, 1994), the idea being that frequently encountered odors are probably referred to with high-frequency words. Again, this assumption has never been explicitly tested. To date, there is no evidence that word frequency of the odor label, rather than familiarity or frequency of occurrence of the odor itself affects odor naming.

In the current study, we investigated to what extent a psycholinguistic variable such as odor label frequency influences odor naming. Based on previous research, we hypothesized odors with high-frequency labels to be named correctly more often than odors with low-frequency labels, even when taking other known factors into account.

## Experiment 1

### Method

#### Participants

Participants were 42 native speakers of Dutch (*M*_age_ = 22.8 years old, *SD* = 3.7, range: 18–37 years, 32 female), who were recruited through the Radboud University participant system. They all gave written consent before the experiment began and were paid €7.50 for their participation.

#### Stimuli

Twenty-four odors (see Appendix Table 10) were selected such that their veridical labels were either high or low frequency. We were restricted in our selection of odors such that the concrete objects were easily administrated as odors as well as differing substantially in lexical frequency. Odors were presented in 30-mL dark glass jars with cotton wool covering the objects so participants could not see them. Log-label frequency was determined by combined occurrences in Dutch CELEX (Burnage, 1990), Dutch SUBTLEX (Keuleers, Brysbaert, & New, 2010), Spoken Dutch (Oostdijk, 2000), and OpenSoNaR corpora (Oostdijk, Reynaert, Hoste, & Schuurman, 2013). The number of occurrences in each corpus was summed and then divided by the total size of the four corpora combined. High-frequency (*M*_log(F)_ = 1.56 per million, *SD* = 0.21) and low-frequency labels (*M*_log(F)_ = 0.14 per million, *SD* = 0.32) differed significantly from each other, *t*(22) = 12.881, *p* < .001, *d* = 5.36.

In addition to word frequency, factors that have been shown to influence word production are word length (Klapp, Anderson, & Berrian, 1973; Meyer, Roelofs, & Levelt, 2003), age of acquisition (Barry, Morrison, & Ellis, 1997; Carroll & White, 1973), and image ability/concreteness (Strain, Patterson, & Seidenberg, 1995). We examined each of these variables too. Word length was operationalized as the number of characters in Dutch spelling. Age of acquisition and concreteness values were taken from data provided by Brysbaert, Stevens, De Deyne, Voorspoels, and Storms (2014). There were no differences in word length *t*(22) = 1.290, *p* = .210, *d* = 0.53, and concreteness *t*(13.511), *p* = .233, *d* = 0.51, between the high-frequency and low-frequency conditions, but age of acquisition differed significantly, *t*(18.795) = 4.89, *p* < .001, *d* = 2.00. However, as all these factors have been shown to be strongly related (Morrison, Chappell, & Ellis, 1997), we focused on the effect of label frequency in the analyses to avoid issues with collinearity (following Baayen, 2008).

#### Procedure

The experiment consisted of two parts: a naming and rating task, followed by a questionnaire, and took approximately 45 minutes to complete.

In the naming/rating task, participants were asked to name the odors by verbally answering the question *Welke geur is dit?* (“Which odor is this?”). Participants were allowed to smell each odor as often as they liked, and were permitted multiple responses. After naming each odor, participants rated it on five different 7-point Likert scales, using Qualtrics Survey Software on a desktop computer. The order of rating scales was identical for all trials and all participants: (1) intensity (how strong the odor smelled), (2) familiarity (how familiar the odor was), (3) pleasantness (how pleasant the odor was), (4) edibility (how edible an object with this odor would be) and, (5) odor frequency (how often the participant personally encountered the odor). High values on the scale stood for high intensity, familiarity, and so forth. Odor presentation order was randomized between participants.

After the main experimental task, participants were asked to complete a demographic questionnaire about their background, including information pertinent to the experiment (e.g., smoking, allergies, illness, cooking experience). All participants were instructed not to eat or smoke at least 1 hour before the experiment.

### Results

#### Odor ratings

Before examining odor naming, participants’ rating scores for odors (see Table 1) were compared to assess potential covariates. Participant fatigue to odors was also assessed by correlating rated intensity with the order in which odors were presented. In addition, we checked correlations between label frequency, familiarity rating, and odor frequency rating to assess whether familiarity and frequency are related and whether familiar odors are indeed described with high-frequency labels.

**Table 1:** Mean ratings (standard deviation in brackets) for the five rating scales for the two subsets of odor stimuli

	High-frequency label	Low-frequency label
Intensity	4.54 (0.71)	5.12 (0.64)
Familiarity	4.60 (0.75)	5.03 (0.72)
Pleasantness	4.11 (0.66)	4.49 (0.69)
Edibility	4.00 (0.97)	4.19 (0.83)
Frequency	3.76 (0.72)	3.97 (0.75)

Odors with low-frequency labels were rated as more intense than those with high-frequency labels, *t*(42) = 7.99, *p* < .001, *d* = 1.23. However, there was no significant correlation between intensity rating and the order in which odors were presented, *r*(22) = .129, *p* = .550, indicating that participants showed no signs of fatigue. Odors with low-frequency labels were rated as more pleasant than odors with high-frequency labels, *t*(42) = 4.42, *p* < .001, *d* = 0.68. There was no difference in edibility ratings between odors with low-frequency or high-frequency labels, *t*(42) = 1.33, *p* = .189, *d* = 0.21.

Odors with low-frequency labels were rated as more familiar than those with high-frequency labels, *t*(42) = 4.42, *p* < .001, *d* = 0.68, and as more frequently occurring, *t*(42) = 2.71, *p* = .010, *d* = 0.41. There was a strong correlation between odor frequency rating and odor familiarity rating, *r*(22) = .945, *p* < .001, indicating these two factors are related. However, there were no significant correlations between familiarity ratings and log-frequency of odor labels, *r*(22) = −.137, *p* = .524, or odor frequency rating and log-frequency of odor label, *r*(22) = −.021, *p* = .923. While participants’ subjective ratings might not reflect true odor frequency in the environment, previous assumptions in the literature that familiar and frequently occurring odors are also described with high frequency *words* (e.g., Jönsson, 2005) is not necessarily true.

As we found significant differences between odors with high-frequency and low-frequency labels, rating scores were also included in the modelling process to assess the contribution of these factors to odor naming.

#### Odor naming

Audio-recordings of participants’ responses on the naming task were transcribed, after which main responses were extracted. Main responses were defined as content responses (without modifiers; e.g., *a bit like beer* was coded as “beer”), excluding hedonic judgments, intensity judgments, and descriptions of elusive sensations (e.g., “familiar”; “recognizable”). For the critical analysis of naming accuracy, only the 24 predefined veridical labels were counted as target answers since their word frequencies and other psycholinguistic variables were considered the point of reference. Results from the naming task were analyzed using mixed logit models (Jaeger, 2008), appropriate for binomially distributed outcomes described as a combination of fixed and random effects. The analyses were done in R (R Core Team, 2013), using the lme4 package (Bates, Maechler, Bolker, & Walker, 2015). In the final model,^1^ log-label frequency was added as fixed factor, with odor familiarity, odor frequency and odor object edibility ratings as covariates.^2^ Including interactions did not significantly improve the model. The dependent variable was naming accuracy (correct; incorrect). We controlled for random participant and item effects. As participants were permitted to give more than one response, separate analyses were conducted for first responses and all responses, to see if effects were stable across time, as Jescheniak and Levelt (1994) found that frequency effects are only detectable in the immediate time frame and disappear soon after. As such, we would expect to find frequency effects in first responses but not necessarily in all responses. For the all-responses analysis, if any of the responses participants gave for a particular odor was the veridical label, this was counted as correct.

**Fig. 1:**
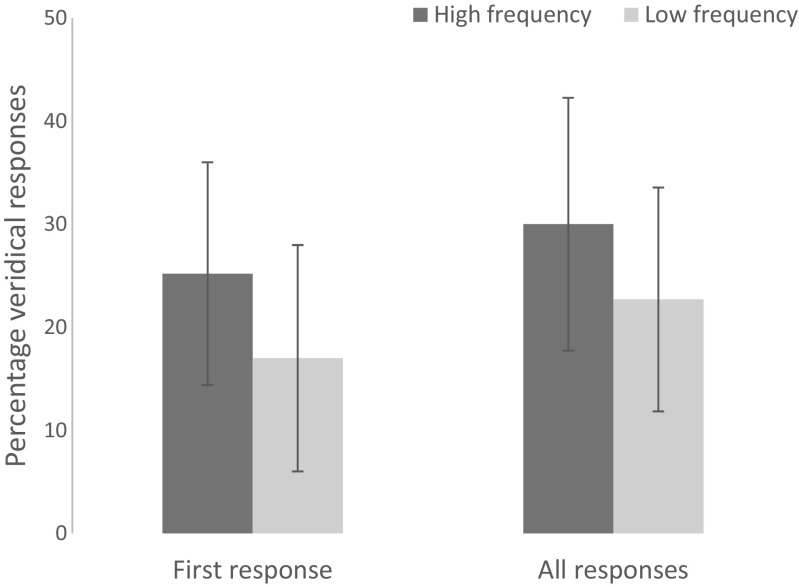
Percentage of veridical answers for first responses and all responses, for the two subsets (high label frequency; low label frequency) of odor stimuli in Experiment 1. Error bars represent standard deviation by participant

For participants’ first responses (see Table 2), properties of the label influenced odor naming, as did properties of the odor itself. Label frequency significantly predicted naming accuracy, β = 1.01, *SE* = 0.51, *z* = 2.01, *p* = .044. Odors with high-frequency labels (25.2%) were named correctly more often than those with low-frequency labels (17.0%); see Fig. 1. In addition, odor familiarity predicted naming accuracy: β = 0.43, *SE* = 0.12, *z* = 3.50, *p* = .001; as did edibility: β = 0.34, *SE* = 0.08, *z* = 4.15, *p* = .001; and rated odor frequency: β = 0.29, *SE* = 0.10, *z* = 2.78, *p* = .005.

**Table 2:** Naming accuracy model for first responses (*N* = 975, log-likelihood = −305.8) in Experiment 1

	Estimate	*SE*	*z*	*p*
(Intercept)	−8.28	0.96	−8.67	.001***
Label frequency	1.01	0.51	2.01	.044*
Odor familiarity	0.43	0.12	3.50	.001***
Odor edibility	0.34	0.08	4.15	.001***
Odor frequency	0.29	0.10	2.78	.005***

* Significant at <.05 level

*** Significant at <.001 level

For all responses (see Table 3), naming accuracy was again predicted by odor familiarity: β = 0.43, *SE* = 0.11, *z* = 3.90, *p* = .001; odor edibility: β = 0.31, *SE* = 0.08, *z* = 4.36, *p* = .001; and odor frequency: β = 0.33, SE = 0.10, *z* = 3.33, *p* = .001. However, the effect of label frequency disappeared, *β* = 0.65, *SE* = 0.40, *z* = 1.63, *p* = .103, even though odors with high-frequency labels (30.0%) were still named correctly more often than those with low-frequency labels (22.7%) numerically; see Fig. 1.

**Table 3:** Naming accuracy model for all responses (*N* = 975, log-likelihood = −348.9) in Experiment 1

	Estimate	*SE*	*z*	*p*
(Intercept)	−7.21	0.79	−9.12	.001***
Label frequency	0.65	0.40	1.63	.103
Odor familiarity	0.43	0.11	3.90	.001***
Odor edibility	0.31	0.08	4.36	.001***
Odor frequency	0.33	0.10	3.33	.001***

* Significant at <.05 level

*** Significant at <.001 level

#### Nonveridical responses

As most responses were nonveridical, it is interesting to look at these in more detail and see whether there are psycholinguistic factors involved in these responses as well. That is, when people are unsure of how to name a smell and have to choose from a set of similar alternative responses, they may resort to higher frequency options. Following Cain (1979), incorrect responses were divided into near misses (the label given was similar to the odor object, e.g., *fennel* for “anise”), and far misses (generic terms, e.g., *spice* for “anise”); and clearly incorrect responses (e.g., *petrol* for “anise”). Two independent judges classified participant’s responses (Cohen’s κ = .938). Disagreements were resolved by discussion, which led to a final list of terms counted as near misses used in the analysis. We expected more near misses for odors with low-frequency labels, as these would undergo more competition from similar responses in the word production process.

We compared the distribution of response types (hit; near miss; far miss) by label frequency (high; low)—see Table 4. There was a significant association between label frequency and response type, both for first responses, *χ*^*2*^(2) = 22.42, *p* < .001, and all responses, *χ*^*2*^(2) = 25.40, *p* < .001. In both cases, the odds of participants responding with a near miss (e.g., *fennel* for “anise”) was 2.73 times higher for odors with low-frequency labels than for odors with high-frequency labels.

**Table 4:** Number of hits, near misses, and far misses as first responses and all responses for odors with high-frequency and low-frequency labels in Experiment 1

		Hit	Near miss	Far miss
High frequency	First response	112	28	376
	All responses	134	35	347
Low frequency	First response	90	72	354
	All responses	113	87	316

## Discussion

Overall, results from the naming task were in line with findings from previous olfaction studies: People seem to perform rather poorly when naming odors. Even when taking all responses into account, participants were, on average, able to correctly name only 26.3% of odors in total. However, the analyses also showed that certain odors were named correctly more easily than others. Factors related to the odor mattered: Odors with higher familiarity, odor frequency, and perceived edibility all contributed to correct naming. Even though the stimuli were chosen based on their widespread availability in the Netherlands—and therefore presumably familiar to Dutch native speakers—familiarity and odor frequency ratings still spanned the entire scale, demonstrating variation, nevertheless. Edibility was not used as a selection criterion in the study design, and most of the odor objects (19 out of 24) were edible. But it seems that edibility plays a role in identifying odors, and subsequently naming them. Some food objects were not recognized as edible: participants gave a food object the lowest edibility rating around 13% of the time. This resulted in misidentification, making *perceived* edibility—rather than generally accepted object edibility—the relevant factor in naming odors.

As predicted, psycholinguistic variables also contributed to correctly naming odors. Most importantly, odors with high-frequency labels were named correctly more often than odors with low-frequency labels upon first response. This confirms the hypothesis that there is a frequency effect at work in odor naming. This study is the first, to our knowledge, to demonstrate a label frequency effect for odor naming. The frequency effect only appeared for first responses, but not for responses produced thereafter, in line with results from picture-naming studies that show the frequency effect is short lived (e.g., Jescheniak & Levelt, 1994). However, this could be considered surprising, as odor perception itself has a longer time course than visual processing of pictures (Keetels & Vroomen, 2012; Khan & Sobel, 2004), which could mean that we would be less likely to uncover frequency effects for odor naming. But this was not the case. Characteristics of the odor label did influence odor naming. We matched odors for the length of their labels as well as concreteness ratings. However, there was a difference in the age of acquisition between conditions that was confounded with frequency. So the results from Experiment 1 could be explained as the result of the frequency of odor labels or the age at which the labels were learned. We come back to this after Experiment 2.

Based on the familiarity and frequency ratings of the two sets of odors (high vs. low label frequency), we believe the differences we find can indeed be attributed to properties of the odor *label* and not to characteristics of the odors themselves. As far as can be determined, the odors in our study were equally discriminable across frequency conditions (cf. Chrea, Valentin, Sulmont-Rossé, Hoang Nguyen, & Abdi, 2005). Some of the odors used in the current study appear in previous tests of odor identification and naming too (e.g., Doty, Shaman, & Dann, 1984; Hummel, Sekinger, Wolf, Pauli, & Kobal, 1997; Kobayashi, Saito, Kobayakawa, Deguchi, & Costanzo, 2006; Cho, Jeong, Lee, Hong, Yoon, & Kim, 2009)—this includes odors with high-frequency labels (coffee, mint, fish) as well as odors with low-frequency labels (anise, cinnamon, turpentine). Importantly, odors with low-frequency labels in our study were rated as both more familiar and more frequently occurring, so properties of the odor are not confounded with odor label properties.

If odor naming is influenced by the odor label (i.e., its frequency), do other psycholinguistic variables also play a role? Aside from word frequency, another factor that influences word production is semantic context. When semantically related pictures (e.g., animals or fruits) are presented consecutively (versus intermingled) for multiple trials, they are named slower (Damian et al., 2001; Kroll & Stewart, 1994), and with increased error rates (Vitkovitch, Humphreys, & Lloyd-Jones, 1993). While this effect has been shown for visual stimuli, we do not know whether it plays a role in other modalities, such as olfaction. It is possible there would be a higher likelihood of interference and problems in naming odors, since the difference between oranges and lemons, for example, is generally perceived to be smaller in smell than in vision (see Schab & Cain, 1991). This is likely to increase competition between related candidates, the cause of semantic interference (Levelt, Roelofs, & Meyer, 1999; Xavier-Alario, Segui, & Ferrand, 2000).

We therefore investigated whether semantic context also influences odor naming. We conducted a second experiment to investigate this issue. The dimensions along which odors are perceived are poorly understood. Even so, edibility is often shown to be important (Ayabe-Kanamura, Kikuchi, & Saito, 1997; Schiffman, Reynolds, & Young, 1981; and Experiment 1 above), so the semantic context we manipulated was edibility. Most semantic interference studies in the visual domain include categories like “food,” “fruit,” or “vegetables” (e.g., Costa, Alario, & Caramazza, 2005; Damian et al., 2001; Jescheniak, Matushanskaya, Mädebach & Müller, 2014; Kroll & Stewart, 1994). Interfering distractors in these studies are always other food items, whereas distractors from other categories are inedible objects. Based on these previous studies, we predicted consecutive odors would be named correctly more often when presented in a semantically unrelated context (i.e., an edible odor followed by an inedible one) than in a semantically related context (e.g., a series of edible odors).

## Experiment 2

### Method

#### Participants

Participants were 40 native speakers of Dutch (*M*_age_ = 24.3 years old, *SD* = 7.3, range: 18–65 years), recruited through the Radboud University participant system. There were 20 participants in each experimental condition, with equal numbers of males and females. Participants gave written consent before the experiment and were paid €15.

#### Stimuli

Twenty-four odors (see Appendix Table 11) were selected such that their veridical labels were either high or low frequency, but also that the words denoted either edible or inedible objects. The odors were presented in 500-mL opaque white plastic squeezy bottles. Participants could not see the odor object but could smell the odor by squeezing the bottle.

As in Experiment 1, log-label frequency was determined by combined occurrences in Dutch CELEX (Burnage, 1990), Dutch SUBTLEX (Keuleers et al., 2010), Spoken Dutch (Oostdijk, 2000), and OpenSoNaR (Oostdijk, Reynaert, Hoste, & Schuurman, 2013) corpora. High-frequency (*M*_log(F)_ = 1.30 per million, *SD* = 0.38) and low-frequency labels (*M*_log(F)_ = −0.23 per million, *SD* = 0.43) differed significantly from each other *t*(22) = 9.29, *p* < .001, *d* = 3.78. Veridical label frequency for edible and inedible odor objects did not differ significantly *t*(22) = 0.92, *p* = .368, *d* = 0.37. Word length (number of characters in Dutch spelling) did not differ significantly for high-frequency versus low-frequency labels *t*(22) = 1.957, *p* = .063, *d* = 0.80, nor for edible versus inedible odor objects *t*(22) = 0.258, *p* = .799, *d* = 0.11. There were also no significant differences in concreteness (Brysbaert et al., 2014) for high-frequency versus low-frequency labels *t*(22) = 2.052, *p* = .052, *d* = 0.84, nor edible versus inedible odor objects, *t*(22) = 1.625, *p* = .118, *d* = 0.66. Age of acquisition of the odor labels (Brysbaert et al., 2014) differed for high-frequency versus low-frequency labels *t*(22) = 5.287, *p* < .001, *d* = 5.37 (as in Experiment 1), but not for edible versus inedible odor objects *t*(22) = 0.168, *p* = .868, *d* = 0.07.

#### Procedure

The experiment consisted of three parts: a naming task, a rating task, and a demographic questionnaire. Participants rated the odors in a separate task this time, increasing the duration of the experiment as well as possible fatigue. We therefore had short breaks of approximately 10 minutes between tasks. The experiment took approximately 75 minutes in total.

In the naming task, participants were asked to name odors as in Experiment 1, with multiple responses permitted. There were two presentation conditions: odors were presented in either a random order or in two blocks of 12 odors based on their edibility. Odor presentation order was randomized between participants.

In the rating task, participants smelled the odors a second time—also in either random or blocked order—and rated the odors on intensity, familiarity, pleasantness, edibility, and odor frequency, as in Experiment 1. The order of rating scales was identical across trials and participants.

After the two main experimental tasks, participants were asked to complete a questionnaire about their personal and linguistic background. Participants were again instructed not to eat or smoke at least 1 hour before the experiment.

### Results

#### Odor ratings

Participants’ rating scores for odors (see Table 5) were compared using a 2 × 2 within-participants ANOVA, with label frequency (high; low) and odor type (edible; inedible) as independent variables, and rating scores as the dependent variables. We also checked correlations between intensity and the order in which odors were presented, and between label frequency, familiarity rating, and odor frequency rating.

**Table 5:** Mean ratings (standard deviation in brackets) for the five rating scales for each subset of odor stimuli

	Food		Nonfood	
	High frequency label	Low frequency label	High frequency label	Low frequency label
Intensity	5.80 (0.61)	5.67 (0.73)	5.61 (0.69)	5.72 (0.61)
Familiarity	5.48 (0.89)	5.71 (0.78)	5.11 (0.90)	5.40 (0,81)
Pleasantness	3.97 (0.91)	4.91 (0.92)	3.16 (0.68)	4.28 (0.79)
Edibility	4.82 (1.34)	5.26 (1.00)	1.57 (0.54)	1.71 (0.66)
Odor frequency	4.30 (1.05)	4.01 (0.95)	3.61 (1.05)	3.91 (0.74)

There was no difference in intensity ratings between food and nonfood odors, *F*(1,39) < 1, *p* = .461, nor between odors with high-frequency labels and low-frequency labels, *F*(1, 39) < 1, *p* = 927. There was also no significant correlation between intensity and the order in which the odor was presented *r*(24) = .200, *p* = .348, meaning participants did not show signs of olfactory fatigue. Food odors were rated as more edible than nonfood odors, *F*(1, 39) = 395.53, *p* < .001, η_p_^2^ = .91, confirming the manipulation was successful. Food odors were also rated as more pleasant than nonfood odors, *F*(1, 39) = 25.22, *p* < .001, η_p_^2^ = .39. Odors with low-frequency labels were rated as more pleasant, *F*(1, 39) = 146.99, *p* < .001, η_p_^2^ = .79, and as more edible than those with high-frequency labels, *F*(1, 39) = 5.03, *p* = .031, η_p_^2^ = .11.

Food odors were rated as more familiar, *F*(1, 39) = 7.51, *p* = .009, η_p_^2^ = .16, and more frequently occurring than nonfood odors, *F*(1, 39) = 8.47, *p* = .006, η_p_^2^ = .18. Odors with low-frequency labels were rated as more familiar than those with high-frequency labels, *F*(1, 39) = 5.23, *p* = .028, η_p_^2^ = 12, but there was no difference in their odor frequency rating, *F*(1, 39) < 1, *p* = .939. There were no significant correlations between familiarity rating and the log-frequency of the odor label, *r*(22) = .06, *p* = .794, or between odor frequency rating and the log-frequency of the odor label, *r*(22) = .28, *p* = .188. However, there was a strong correlation between odor frequency rating and odor familiarity rating, *r*(22) = .75, *p* < .001.

As Experiment 2 used different stimuli, some of the rating scores (intensity, odor frequency) differed compared to Experiment 1. Nevertheless, findings from the rating task were largely in line with what we found in the first experiment, and as such, rating scores were again included in the modelling process.

#### Odor naming

The data were coded and analyzed as in Experiment 1. In the final model,^3^ log-label frequency and semantic context (random; blocked) were added as fixed factors, with familiarity and edibility ratings as covariates. Including interactions did not significantly improve the model. The dependent variable was naming accuracy (correct; incorrect). We controlled for random participant and item effects.

**Fig. 2:**
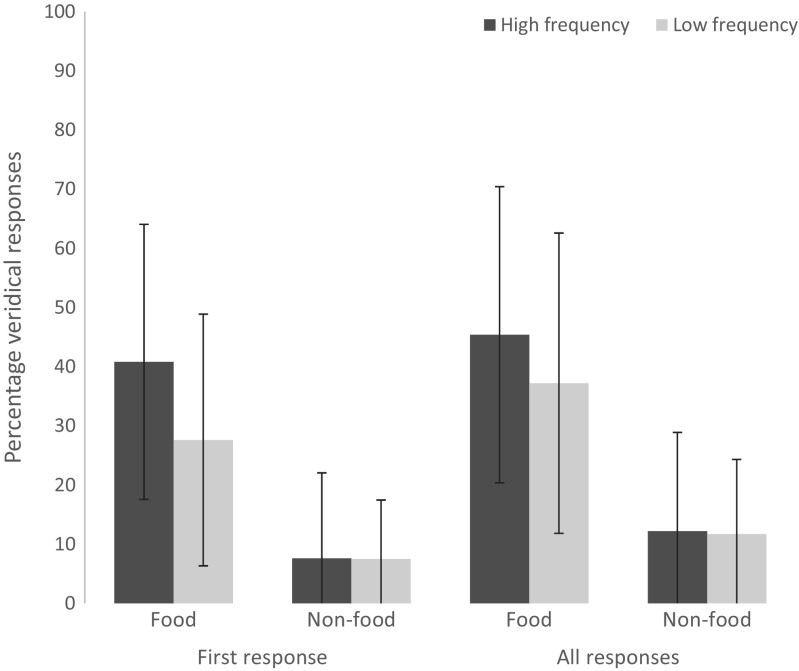
Percentage of veridical answers in Experiment 2 for first responses and all responses, plotted by food and nonfood items; high and low label frequency. Error bars represent standard deviation by participant

For participants’ first responses (see Table 6), label frequency significantly predicted naming accuracy, β = 0.71, *SE* = 0.29, *z* = 2.44, *p* = .014. Odors with high-frequency labels (24.3%) were named correctly more often than those with low-frequency labels (17.6%). However, there was no difference in naming accuracy between the random (22.5%) and blocked (19.3%) semantic context conditions, β = −0.08, *SE* = 0.27, *z* = 0.30, *p* = .761; see Fig. 2. In addition, familiarity predicted naming accuracy: β = 0.57, *SE* = 0.11, *z* = 5.47, *p* < .001, as did edibility: β = 0.24, *SE* = 0.06, *z* = 3.86, *p* < .001.

**Table 6:** Naming accuracy model output (*N* = 975, log-likelihood = −346.9)

	Estimate	*SE*	*z*	*p*
(Intercept)	−6.55	0.74	−8.88	<.001***
Semantic context	−0.08	0.27	−0.30	.76
Label frequency	0.71	0.29	2.44	.01*
Odor familiarity	0.57	0.11	5.47	<.001***
Odor edibility	0.24	0.06	3.86	<.001***

* Significant at <.05 level

*** Significant at <.001 level

For all responses (see Table 7), odors with high-frequency labels (28.9%) were also named correctly more often than those with low-frequency labels (24.4%) in all responses, β = 0.51, *SE* = 0.24, *z* = 2.14, *p* = .033. But again, there was no difference in naming accuracy between random (28.2%) and blocked (25.2%) conditions, β = −0.06, *SE* = 0.26, *z* = 0.24, *p* = .81; see also Fig. 2. There was also an effect of familiarity: β = 0.47, *SE* = 0.08, *z* = 5.60, *p* < .001, and edibility: β = 0.26, *SE* = 0.05, *z* = 4.84, *p* < .001.

**Table 7:** Naming accuracy model output (*N* = 975, log-likelihood = −406.8)

	Estimate	*SE*	*z*	*p*
(Intercept)	−5.27	0.58	−9.11	<.001***
Semantic context	−0.06	0.26	−0.24	.81
Label frequency	0.51	0.24	2.14	0.03*
Odor familiarity	0.47	0.08	5.60	<.001***
Odor edibility	0.26	0.05	4.84	<.001***

* Significant at <.05 level

*** Significant at <.001 level

#### Nonveridical responses

As in Experiment 1, incorrect responses were divided into hits, near misses (e.g., *fennel* for “anise”) and far misses (generic terms and clearly incorrect responses) by two independent judges (Cohen’s κ = .931). Disagreements were resolved by discussion.

We compared the distribution of response types (hit; near miss; far miss) by label frequency (high; low). In the random-order condition (see Table 8), there was a significant association between label frequency and response type, *χ*^*2*^(2) = 13.46, *p* < .005. Upon first response, the odds of participants responding with a near miss (e.g., *fennel* for “anise”) were 2.29 times higher for odors with low-frequency labels than for odors with high-frequency labels. The same pattern appeared when taking all responses into consideration, but this was not significant at the conventional level of significance *χ*^*2*^(2) = 5.46, *p* = .065.

**Table 8:** Random order condition: Number of hits, near misses, and far misses as first responses and all responses for odors with high-frequency and low-frequency labels

		Hit	Near miss	Far miss
High frequency	First response	63	25	132
	All responses	73	44	123
Low frequency	First response	45	53	141
	All responses	62	65	112

In the blocked condition (see Table 9), however, there was no significant association between label frequency and the type of response examining first responses, *χ*^*2*^(2) = 3.26, *p* = .2, or all responses, *χ*^*2*^(2) = 1.36, *p* = .51.

**Table 9:** Blocked order condition: Number of hits, near misses, and far misses as first responses and all responses for odors with high-frequency and low-frequency labels

		Hit	Near miss	Far miss
High frequency	First response	53	37	147
	All responses	65	56	116
Low frequency	First response	39	46	154
	All responses	55	52	126

## General discussion

In both Experiment 1 and 2, we found a robust frequency effect demonstrating properties of the label matter for odor naming. At the same time, there was little evidence of semantic interference for odor naming. Blocking odors by edibility led to numerically lower correct naming (28% for random order vs. 25% for blocked order), but this was not statistically significant. As mentioned in the discussion of Experiment 1, the dimensions of odor perception are poorly understood, which is why we chose to investigate fairly broad semantic categories. This is in line with previous picture-naming studies that also used superordinate categories, such as animals. However, it is possible stimuli from such broad semantic categories are too diverse to adequately capture semantic interference in odor naming. Even so, semantic categories that have been used in picture naming and word naming studies (e.g., animals, tools, professions) are usually not suitable for odor experiments, as most of the items in these groupings do not typically have an odor, so this remains a challenge for future work along these lines.

We do not believe the lack of a semantic interference effect is due to a failure of odor identification. Although we only test naming ability, interference effects are predicted to come from *edibility* characteristics of the odor object, and the rating data from Experiment 2 show that participants have no problem judging edibility. This is in line with previous research (Fusari & Ballesteros, 2008) and means that, in principle, the experimental manipulation in itself should have worked.

There are differences in production between semantically related words (e.g., *lemon* and *orange*) and associatively related words (e.g., *lemon* and *juice*). Whereas semantically related words suffer from interference in production, associatively related words enjoy facilitation (e.g., Xavier-Alario et al., 2000). As there was no difference between conditions, it might be the case that some labels were both semantically and associatively related (e.g., *coffee* and *tea*), thereby neutralizing interference and facilitation effects. If there are interference or facilitation effects at work in odor naming, they might be revealed with a more limited set of odors specifically selected for either semantic relatedness or associative relatedness, with label frequency and other psycholinguistic variables balanced.

Analysis of nonveridical responses also revealed some interesting patterns. In some cases, responses were actually more specific than the predetermined veridical labels (e.g., *green tea* instead of just *tea*), which might reflect the speaker’s intention to be as informative as possible (Grice, 1975; Levelt, 1996). In others, a particular response classified as a near miss (erroneous, yet appropriate) was used by a large number of participants. Overall, odors with low-frequency labels were named with a near miss label more often than odors with high-frequency labels. For example, we used bleach to represent *chloor* (chlorine) in Experiment 1, a label with a higher word frequency (log(F) = 0.13 per million) than the actual source term *bleekmiddel* (log(F) = −0.65 per million; veridical in Experiment 2). In the two experiments combined, c*hloor* was used by 53 out of 83 participants, whereas *bleekmiddel* was used by only three participants. This suggests predetermined target labels (e.g., based on the odor source) might not always be considered the conventional label by the speech community (cf. Dubois, 2000), who instead might opt for the an alternative, frequently occurring label, if it describes the odor adequately.

In these studies, we focused on the role of odor label frequency on odor naming. However, a number of psycholinguistic variables correlate strongly with one another: high-frequency words tend to be shorter, are acquired earlier, and are higher in concreteness (e.g., Brysbaert et al., 2014). Indeed, in both Experiment 1 and Experiment 2, high-frequency and low-frequency conditions also differed in age of acquisition, although not in length or concreteness. So, the current studies leave open the possibility that the psycholinguistic effects demonstrated herein are related to age of acquisition of words as well as frequency. Ultimately, both are likely to be of importance (cf. Brysbaert, Lange, & Van Wijnendaele, 2010).

Overall, then, the results of this study demonstrate that it is important to consider properties of the lexicon, alongside properties of odors, when investigating olfactory language and cognition. These results have broader implications too. As mentioned, Majid and Burenhult (2014) showed there is a difference in the odor-naming ability between speakers of Jahai and English. The current study sheds possible new light on why this difference exists; Perhaps Jahai speakers talk about odors more frequently than English speakers do. There is no direct evidence for this proposal, but San Roque et al. (2015) compared the frequency of perception verbs (e.g., *look, hear, touch, taste, smell*) in 13 diverse languages and found smell verbs were more frequent in Semai (a language closely related to Jahai) than in any other language, suggesting that smell is talked about more often. So, in principle, word frequency is a possible proximate explanation for why smells are easier to name for the Jahai, and, conversely, more difficult to name for speakers of Standard Average European languages (cf. Köster, Møller, & Mojet, 2014).

Moreover, various olfaction tests have been used in clinical settings—for example, the University of Pennsylvania Smell Identification Test (UPSIT]; Doty et al., 1984) and the Sniffin’ Sticks Identification Test (Hummel et al., 1997), and efforts have been made to create culturally appropriate tests as well (e.g., the Odor Stick Identification Test for Japanese [OSIT-J]: Saito et al., 2006; Barcelona Smell Test–24 [BAST-24]: Cardesín et al., 2006; Italian Olfactory Identification Test [IOIT]: Maremmani et al., 2012). Such tests often use a forced-choice format, where the participant has to choose which of, for example, four different labels applies to an odor. Much consideration has been given to how the test set is constructed because it is known that people make more errors when the alternate choices come from a related rather than an unrelated semantic category (e.g., Engen, 1987; Goubet, McCall, Ducz, & Bingham, 2014), and it has been postulated such errors might even have a chemical basis, since related entities may share chemical compounds (Fjaeldstad, Peterson, & Oversen, 2017). Now that we have shown that properties of the lexicon play a role in odor naming, such tests can be further improved by taking these results into consideration so as to enable more control over inadvertent factors that influence test performance. For example, high-frequency labels may become inadvertent false lures in such tests; and when tests are translated from language to language, descriptors ought to be matched on psycholinguistic variables so as to avoid inadvertent confounds (cf. Fjaeldstad et al., 2017).

To conclude, we show that odor naming is influenced by word frequency—a factor previously ignored in the olfactory literature—and, at the same time, demonstrate that frequency effects are relevant beyond picture naming, the mainstay of the language production literature. So properties of the odor label are just as important to consider as properties of the odor itself in olfaction research; and psycholinguists should consider how language interfaces with all perceptual modalities, not just vision (Levinson & Majid, 2014).

## Appendix 1

**Table 10.** List of odor stimuli used in the study (split by label frequency), with their veridical labels and log word frequency per million (log(F)/10^6^) in Dutch

Frequency	Odor	Veridical label	log(F)/10^6^	Odor source
High frequency label	Apple	*appel*	1.39	Apple sauce
	Beer	*bier*	1.71	Pilsner beer
	Cheese	*kaas*	1.40	Cheddar cheese
	Chocolate	*chocola*	1.25	Cocoa powder
	Coffee	*koffie*	1.82	Ground coffee
	Fish	*vis*	1.79	Canned fish
	Grass	*gras*	1.38	Fresh grass
	Milk	*melk*	1.49	Milk
	Mint	*munt*	1.46	Mint leaves
	Oil	*olie*	1.63	Motor oil
	Wine	*wijn*	1.90	Red wine
	Wood	*hout*	1.50	Cedar wood
Low frequency label	Anise	*anijs*	−0.38	Star anise
	Chlorine	*chloor*	0.13	Bleach
	Cinnamon	*kaneel*	0.26	Ground cinnamon
	Coconut	*kokos*	0.20	Coconut jam
	Cork	*kurk*	0.39	Cork essence
	Kiwifruit	*kiwi*	0.32	Fresh kiwifruit
	Leek	*prei*	0.48	Fresh leek
	Lime	*limoen*	0.08	Lime juice
	Mango	*mango*	0.44	Fresh mango
	Pine	*denne*	−0.03	Pine essential oil
	Sesame	*sesam*	−0.49	Sesame oil
	Thyme	*tijm*	0.35	Fresh thyme

*Note*. Word frequency is based on combined occurrences in the Dutch CELEX, Dutch SUBTLEX, Spoken Dutch, and OpenSoNaR corpora

## Appendix 2

**Table 11.** List of odor stimuli used in the study (split by label frequency and edibility), with their veridical labels and log word frequency per million (log(F)/10^6^) in Dutch

Frequency	Edibility	Odor	Veridical label	log(F)/10^6^	Odor source
High frequency label	Food	Beer	*bier*	1.71	Pilsner beer
		Chocolate	*chocola*	1.25	Cocoa powder
		Coffee	*koffie*	1.82	Ground coffee
		Mint	*munt*	1.46	Mint leaves
		Potato	*aardappel*	1.23	Mashed potatoes
		Tea	*thee*	1.39	Earl Grey
	Nonfood	Ash	*as*	1.76	Cigarette ash
		Matches	*lucifers*	1.05	Struck matches
		Petrol	*benzine*	1.09	Unleaded petrol
		Rose	*roos*	1.42	Rose essential oil
		Soap	*zeep*	0.98	Soft soap
		Tobacco	*tabak*	0.88	Rolling tobacco
Low frequency label	Food	Anise	*anijs*	−0.38	Star anise
		Cinnamon	*kaneel*	0.26	Ground cinnamon
		Coconut	*kokos*	0.20	Coconut oil
		Nutmeg	*nootmuskaat*	0.06	Ground nutmeg
		Oregano	*oregano*	−0.28	Ground oregano
		Sesame	*sesam*	−0.49	Sesame oil
	Nonfood	Bleach	*bleekmiddel*	−0.65	Bleach
		Detergent	*waspoeder*	−0.12	Detergent
		Incense	*wierook*	0.31	Church incense
		Pine	*denne*	−0.03	Pine essential oil
		Turpentine	*terpentine*	−0.63	Turpentine
		Ylang	*ylang*	−1.18	Ylang essential oil

*Note*. Word frequency is based on combined occurrences in the Dutch CELEX, Dutch SUBTLEX, Spoken Dutch, and OpenSoNaR corpora

## Footnotes

1 The initial model also included odor intensity and odor pleasantness. However, these factors did not significantly improve the model, all *p*s > .05.

2 Odor familiarity and odor frequency were strongly correlated, but both factors were included following Wurm & Fisicaro (2014).

3 The initial model also included odor intensity, odor pleasantness, and odor frequency. However, these factors did not significantly improve the model, all *p*s > .05.
